# Optimization of Multistage Extraction of Olive Leaves for Recovery of Phenolic Compounds at Moderated Temperatures and Short Extraction Times

**DOI:** 10.3390/foods3010066

**Published:** 2013-12-30

**Authors:** Konstantinos Stamatopoulos, Archontoula Chatzilazarou, Evangelos Katsoyannos

**Affiliations:** 1 Department of Food Technology, Faculty of Food Technology and Nutrition, Technological Educational Institute of Athens, 12 Ag. Spyridonos St., Egaleo, Athens 122 10, Greece; E-Mail: stamato_k@hotmail.com; 2 Department of Oenology and Beverage Technology, Faculty of Food Technology and Nutrition, Technological Educational Institute of Athens, 12 Ag. Spyridonos St., Egaleo, Athens 122 10, Greece; E-Mail: arhchatzi@yahoo.gr; 3 Department of Food Technology, Faculty of Food Technology and Nutrition, Technological Educational Institute of Athens, 12 Ag. Spyridonos St., Egaleo, Athens 122 10, Greece

**Keywords:** polyphenols, antioxidant activity, multistage extraction, steam blanching, olive leaf

## Abstract

The aim of the present study was to improve the recovery of polyphenols from olive leaves (OL) by optimizing a multistage extraction scheme; provided that the olive leaves have been previously steam blanched. The maximum total phenol content expressed in ppm caffeic acid equivalents was obtained at pH 2, particle size 0.315 mm, solid-liquid ratio 1:7 and aqueous ethanol concentration 70% (v/v). The optimum duration time of each extraction stage and the operation temperature, were chosen based on qualitative and quantitative analysis of oleuropein (OLE), verbascoside, luteolin-7-*O*-glucoside and apigenin-7-*O*-glucoside performed by high performance liquid chromatography with diode array detector (HPLC-DAD). The optimum conditions for multistage extraction were 30 min total extraction time (10 min × 3 stages) at 85 °C. The 80% of the total yield of polyphenols was obtained at the 1st stage of the extraction. The total extraction yield of oleuropein was found 23 times higher (103.1 mg OLE/g dry weight (d.w.) OL) compared to the yield (4.6 mg OLE/g d.w. OL) obtained by the conventional extraction method (40 °C, 48 h). However, from an energetic and hence from an economical point of view it is preferable to work at 40 °C, since the total extraction yield of polyphenolic compounds was only 17% higher for a double increase in the operating temperature (*i.e.*, 85 °C).

## 1. Introduction

By-products and wastes from plant food processing, which represent a major environmental problem in Mediterranean countries, are sources of added value bioactive compounds called phytochemicals or secondary metabolites [1]. Thus, several studies have focused on the composition of the extracts and improvement processes for maximum recovery of such antioxidant substances [2,3].

Among the different parts of the olive tree, olive leaves possess the highest oleuropein content, within a range of 1%–14% compared to olive oil (0.005%–0.12%) and olive mill wastewater (0.87%) [4]. Several studies have revealed that the health-promoting properties of virgin olive oil are mainly due to the presence of polyphenolic compounds [5]. Oleuropein and related phenolic compounds (e.g., luteolin-7-glucoside, apigenin-7-glucoside, hydroxytyrosol and rutin) have shown cardiovascular protective effects [6,7]. A possible explanation for these effects of olive leaves polyphenols has been attributed to the synergistic phenomena among the phenolic compounds [8].

Several conventional extraction techniques have been reported for the recovery of target compounds from raw materials. Hot water technology is the main and most common extraction method for flavone glycosides and other antioxidants [9,10,11,12]. Methanol-water mixtures [13] have been used in conventional solid-liquid extraction methods for the isolation of phytochemicals. However, the use of non-toxic solvents is preferable for natural extracts production since it leads to the development of functional foods with health-promoting properties.

Numerous extraction-assisted techniques have been developed, such as extraction with superheated liquids [4] ultrasound-assisted extraction [14] and microwave-assisted extraction [15]. Alternatives to those methods have been reported, like supercritical fluid extraction, with CO_2_ as supercritical fluid and ethanol or methanol as modifier [16]. However, it should be pointed out that most of these techniques suffer from high energy costs as they operate under high pressure [17].

Most of the studies, dealing with the optimization of extraction of phenolic compounds from plant sources, have approached this issue mainly from a scientific rather than an industrial point of view. Thus, a single-stage extraction process is the main protocol that is followed by several authors which requires long extraction times (5–24 h) for complete recovery of phenolic compounds [18,19] or sophisticate techniques to improve its efficiency at shorter times. Production of natural antioxidant compounds at industrial scale requires accelerated extraction processes, small extractant volumes, non-toxic solvents, low energy consumption and technical simplicity. Moreover, a new extraction process should be easily adapted on the existed production line of industries.

The optimization of the extraction of phenolic compounds has been focused mainly on single-stage scheme rather than on multistage one. Thus, the authors propose low extraction temperatures (e.g., 40 °C) for high extraction times. However, from an economical point of view it would be advisable to work, e.g., at 60 °C for shorter times [20].

To fulfill the requirements of industry for mass production of natural antioxidant extracts, multi-stage extraction has been proven to be more effective than single step extraction when using equal solvent volume [21]. In this sense, it is preferable to split the total extractant volume to several portions, by carrying out three or more extraction steps [21]. Moreover, Stamatopoulos, Katsoyannos, Chatzilazarou and Konteles [22] showed that steam blanching of olive leaves as pre-treatment of olive leaves extraction significantly improves the extractability of phenolic compounds about 25–35 times and increased the antioxidant activity about 4–13 times of olive leaf extract.

In the literature there is no study, to our knowledge, approaching the improvement of extraction of olive leaves by optimizing the multistage extraction of thermal treated olive leaves. Thus, the aim of this study was to associate steam blanching of olive leaves and multistage extraction scheme for satisfactory recovery of polyphenolic compounds, operating at moderated temperatures and short extraction times.

## 2. Experimental Section

### 2.1. Chemicals

Methanol, acetic acid and acetonitrile were purchased from Merck (Darmstadt, Germany), tyrosol, caffeic acid and 2,2-diphenyl-1-picrylhydrazyl reagent (DPPH) were purchased from Sigma-Aldrich (Hohenbrunn, Germany). Oleuropein and hydroxytyrosol were purchased from Extrasyntese (Genay, France). Sodium acetate trihydrate was purchased from Carlo Erba Reactivs SDS (Val de Reuil, France), rutin from Sigma (St. Louis, MO, USA) while Folin-Ciocalteau reagent, disodium hydrogen phosphate and potassium chloride from Merck (Darmstadt, Germany) and ethanol absolute from Sigma-Aldrich (St. Louis, MO, USA). Apigenin-7-*O*-glucoside, luteolin-7-*O*-glucoside and verbascoside were obtained from ExtraSynthese (Genay, France).

### 2.2. Steam Blanching of Olive Leaves

Prior to multistage extraction process, olive leaves had been processed according to Stamatopoulos, Katsoyannos, Chatzilazarou, and Konteles [22]. Briefly, olive leaves were steam blanched for 10 min in a household steam cooker at atmospheric pressure and immediately they were cooled down by cold water at 17 °C. The excess water was removed by an absorbent paper and subsequently the olive leaves were dried in an air oven for 4 h at 60 °C.

### 2.3. Optimization of Phenolic Compounds Extraction from Olive Leaves

Optimization of extraction of polyphenols from olive leaves was performed in term of particle size, pH, composition of aqueous ethanol solution (v/v) and solid-to-liquid ration, on steam blanched olive leaves. Briefly, fresh olive leaves were dried with an air tray oven at 60 °C for 4 h.

#### 2.3.1. Effect of Particle Size

The dried olive leaves were ground and sieved. Thus, different particle size fractions were obtained, 0.05, 0.1, 0.2, 0.315 and 1.0 mm. Each fraction was extracted separately for 2 h under stirring (400 rpm) at the following conditions: 20% aqueous ethanol solution, solid-solvent ratio 1:10, pH 3 at 40 °C temperature. Then, the samples were centrifuged at 6000 rpm for 5 min. All ethanolic extracts were filtered through 0.45-μm syringe filters and were analyzed for total phenol content by (TPC) Folin-Ciocalteau assay.

#### 2.3.2. Effect of pH

Dried olive leaves were extracted for 2 h at 40 °C, particle size 1 mm and solid-solvent 1:10 with 20% aqueous ethanol solution adjusted at pH 1.3, 2.0 and 3.0 with 0.1 M KCl/HCL buffer solution, at pH 4.2, 5.2 and 6.5 with 0.1 M sodium acetate/acetic acid buffer solution and at pH 8.3 with 0.1 M disodium hydrogen phosphate/HCl buffer system. Each sample was centrifuged at 6000 rpm for 5 min. All ethanolic extracts were filtered through 0.45-μm syringe filters and were analyzed for total phenol content by Folin-Ciocalteau assay.

#### 2.3.3. Effect of Solid-to-Liquid Ratio (S/L)

Dried olive leaves were extracted for 2 h at 40 °C, particle size 1 mm, pH 3 and 20% aqueous ethanol solution in different solid-liquid ratios 1:5, 1:6, 1:7, 1:8 and 1:10. Each sample was centrifuged at 6000 rpm for 5 min. All ethanolic extracts were filtered through 0.45-μm syringe filters and they were analyzed for total phenol content by Folin-Ciocalteau assay. The particle size of 1 mm was preferred because, based on our experience using lower particle size causes problems at low solid-to-liquid ratio (*i.e.*, <1:7), since the mixture of olive leave and solvents became like sludge and hence mixing with magnetic stirrer was problematic.

#### 2.3.4. Effect of Ethanol Concentration (% EtOH, v/v)

Dried olive leaves were extracted for 2 h at different aqueous ethanol concentration of 20%, 40%, 55%, 70%, 80% and 90% (v/v) with the rest parameters to be as followed: 40 °C, 1:10 solid-solvent ratio, 1 mm particle size and pH 3. Each sample was centrifuged at 6000 rpm for 5 min. All ethanolic extracts were filtered through 0.45-μm syringe filters and they were analyzed for total phenol content by Folin-Ciocalteau assay.

### 2.4. Multistage Extraction Scheme of Olive Leaves

After choosing the optimum conditions of extraction in terms of particle size, pH, S/L and % EtOH, a multistage extraction scheme was set up for maximum recovery of polyphenols at moderated to high temperatures and short extraction times. In order to illustrate the significance of combining steam blanching and multistage extraction process, steamed and non-steamed olive leaves were treated, based on multistage extraction protocol. Multistage extraction was performed at different temperatures, 40 °C (mainly recommended in literature), 60, 65, 70 and 85 °C; the rest parameters (pH, S/L, % EtOH) were set up based on the results of the optimization of the single stage extraction experiments (Section 2.3). Moreover, three extraction stages were performed with 60 min duration time per stage. At each stage, sample was collected every 10 min. At the end of every stage, the sample was centrifuged (6000 rpm for 5 min) and the residue was re-extracted with renewal solvent. All the process was repeated three times. Finally, non-steam blanched olive leaves were extracted at 40 °C followed a multistage extraction and the results were compared with the corresponding of steam blanched olive leaves obtained at the same temperature. All ethanolic extracts were filtered through 0.45-μm syringe filters and they were analyzed by HPLC-DAD for qualitative and quantitative analysis of oleuropein, verbascoside, luteolin-7-*O*-glucoside and apigenin-7-*O*-glucoside.

### 2.5. Conventional Extraction Protocol

A procedure was developed using the same extractant (70% v/v aqueous ethanol) and sample-extractant volume ratio (1:7), as used in the multistage extraction scheme, for a time enough to ensure complete extraction of the target analytes and at mild temperature to avoid potential degradation. Dried and milled steamed and non-steamed leaves were immersed separately in 70% EtOH at 1:7 ratio and were placed in a beaker and subjected to stirring at 40 °C for 48 h. The process was repeated three times. Then, all ethanolic extracts were filtered through 0.45-μm syringe filters and they were analyzed by HPLC-DAD for qualitative and quantitative analysis of oleuropein, verbascoside, luteolin-7-*O*-glucoside and apigenin-7-*O*-glucoside.

### 2.6. Chromatographic Conditions

The equipment utilized was a HITACHI (Tokyo, Japan) coupled to an auto-sampler L-2200, pump L-2130, column oven L-2300 and diode array detector L-2455 and controlled by MerckAgilent EZChrom Elite software (Agilent Technologies, Santa Clara, CA, USA). The column was a Pinnacle II RP C18, 3 μm, 150 mm × 4.6 mm (Restek, Bellefonte, PA, USA), protected by a Kromasil 100-5 C18 guard cartridge starter kit (Sigma-Aldrich, Seelze, Germany) for 3.0/4.6 mm i.d. column oven was set at 40 °C. Eluent (A) and (B) were 0.02 M sodium acetate adjusted to pH = 3.2 with acetic acid and HPLC grade acetonitrile, respectively. The flow rate was 1 mL/min and the injection volume 20 μL. The elution gradient profile was as follows: started (A) 90%; 2 min, 85%; 9 min, 75%; 12 min, 65%; 15 min, 55%; 18 min, 40%; 20 min, 90%. Eluting was monitored at 280 nm for oleuropein, hydroxytyrosol and tyrosol and at 355 nm for flavonols.

### 2.7. Determination of Antioxidant Activity

Antiradical activity measurement was performed by using 2,2,-diphenyl-2-picryl-hydrazyl (DPPH) assay according to Braca [23], with some modifications. 2.5 mg of DPPH powder was diluted in 100 mL pure methanol with absorption 0.7 (±0.03) at 517 nm. A sample of every stage at each temperature was diluted 50 times and then 33 μL of the diluted sample was added to an aliquot of 1 mL of 0.004% DPPH solution. To control, 33 μL of pure ethanol were added instead of olive leaf extract. The reaction mixture was vortex-mixed and was allowed to stand in the darkness at room temperature for 30 min before measuring the decrease in absorbance at 517 nm. The spectrophotometer (SHIMADZU mini 1240 UV-Vis, Shimadzu, Columbia, MD, USA) was calibrated with pure methanol. Antioxidant activity (*AA*) was expressed in percentage inhibition of DPPH radical and was calculated with the following equation:

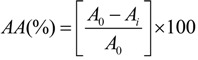
(1)
*A*_0_ and *A_i_* are the absorbance of the control sample and the sample containing olive leave extract respectively. The procedure was repeated three times for each sample.

### 2.8. Determination of Total Phenol Content (TPC)

TPC was determined according to the Folin-Ciocalteau assay method [24] with some modifications. Briefly, all the samples were diluted with ethanol (1:1). Then, 0.1 mL of the diluted sample was put into a 20 mL test tube, into which distilled H_2_O was added to have a final volume of 6.75 mL. Folin-Ciocalteau phenol reagent (0.25 mL) was added to the mixture and shaken vigorously. After 3 min, 3 mL of 35% Na_2_CO_3_ solution was added with mixing. Ethanol (0.1 mL) was used as blank instead of diluted sample. The solution was allowed to stand for 60 min. After this time, the absorbance was measured at 750 nm in comparison the prepared blank. The TPC of sample was expressed as mg caffeic acid equivalents (CAE)/g d.w. OL. The procedure was repeated three times for each sample.

## 3. Results and Discussion

This study attempted to approach the procedure of extraction of olive leaves considering the basic requirements of the mass production of nutraceuticals in industrial scale. Thus, a combination of the widely used thermal treatment (steam blanching) in food industry with a multistage extraction scheme was applied. In preliminary experiments the total phenol content (TPC) of ethanolic extracts of olive leaves was determined with Folin-Ciocalteau assay and HPLC-DAD. It should be noted that when the extraction temperature was ≤40 °C there were no qualitative changes of the phenolic profile of the extracts and hence Folin-Ciocalteau assay was preferred as an easy and fast procedure for the optimization of extraction in terms of pH, particle size, solid-to-liquid ratio (S/L) and ethanol concentration (% EtOH, v/v). However, above that temperature and particular ≥60 °C, the phenolic profile was changed and high amounts of hydroxytyrosol were appeared in the chromatograms. Moreover, the pH (except the sample of 1.3), particle size, solid-to-liquid ratio (S/L) and ethanol concentration (% EtOH, v/v) did not affect significantly the phenolic profile when the extraction temperature was below or equal to 40 °C. Thus, HPLC-DAD was preferred for the optimization of multistage extraction in terms of operation temperature and duration time while the rest parameters were kept constant.

In conclusion, the total phenol content of the extracts (TPC) obtained with Folin-Ciocalteau assay was chosen as a criterion for selecting the optimum conditions of the extraction of olive leaves in terms of pH, particle size, solid-to-liquid ratio (S/L) and ethanol concentration (% EtOH, v/v). Whereas, qualitative and quantitative analyses of oleuropein, verbascoside, luteolin-7-*O*-glucoside and apigenin-7-*O*-glucoside were performed with HPLC in order to evaluate the performance of the multistage extraction *versus* the conventional method (one stage, 48 h, 40 °C) as well as the impact of the applied temperature on the degradation of phenolics during the multistage extraction.

### 3.1. Effect of Particle Size

**Figure 1:**
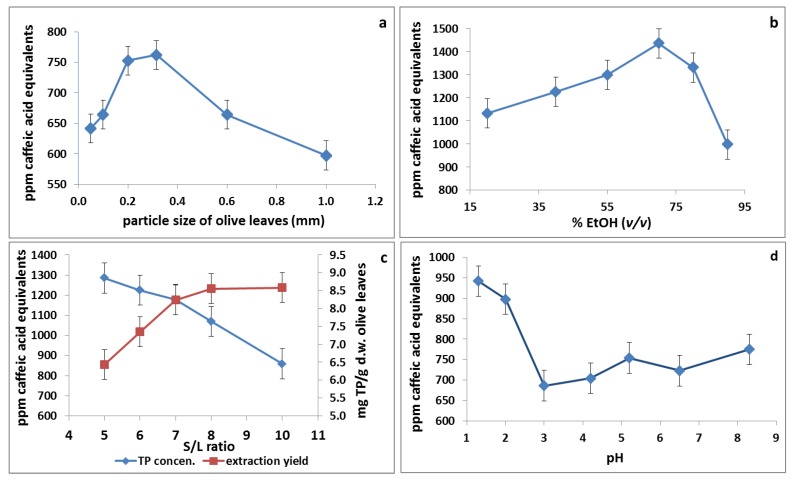
Total phenols content expressed as ppm caffeic acid equivalents, in ethanolic extract of steamed olive leaves as a function of (**a**) particle size of olive leaves (OL); (**b**) solvent composition (v/v); (**c**) solid-to-liquid ratio; and (**d**) pH. The extraction was performed at 50 °C for 2 h under stirring (*n* = 400 rpm).

In this study, the effect of particle size was examined within a range of 0.05–1.0 mm. It was expected that the smallest particle size of olive leaves leads to highest extraction yield. However, for particle size below 0.2 mm the extraction yield is decreased (Figure 1a). This is due to the fact that the particle size has to be limited because exceedingly small particles tend to agglomerate, leading to a decrease of solvent penetration in the solid matrix and, therefore, negatively affecting the mass transfer process [25]. Thus, a typical value of 0.3 mm is accordingly recommended.

### 3.2. Effect of pH

Concerning the recovery of polyphenols, pH can impact according to different mechanisms on the recovery of polyphenols such as increase of solubility of the solute and alternation of the interactions of antioxidants with other constituents of the plant material [25]. Based on our results, the highest extraction yield was obtained at pH 1.3 (Figure 1d). Nevertheless, HPLC-DAD analysis of the current sample showed increased values of hydroxytyrosol due to hydrolysis of oleuropein [26]. Hence, the total phenol content seems higher, although, this is not due to the performance of the extraction at that pH value but due to higher reactivity of the hydroxytyrosol with Folin-Ciocalteau assay. Thus, pH 2.0 seems preferable since at that pH value no hydrolysis of oleuropein was observed. The proposed pH is similar to the value that Mylonaki, Kiassos, Makris, and Kefalas [19] suggest for the extraction of olive leaves. According to Japón-Luján, Luque-Rodríguez, and Luque de Castro [15], the largest amount of oleuropein extracted from olive leaves was found at pH 7; however, the authors did not work at pH values below 3. In conclusion, multistage extraction experiments should be conducted at pH 2 in order to avoid chemical hydrolysis of polyphenols at higher temperatures.

### 3.3. Effect of Solid-to-Liquid Ratio (S/L)

The solvent-to-solid ratio on the recovery of phenolic compounds should be carefully analysed and optimized as the solvent consumption exerts a direct influence on the extraction process cost.

Figure 1c illustrates the effect of S/L on the concentration of total phenolics in the extract (ppm CAE) and the extraction yield (mg CAE/g d.w. OL). The results show that the concentration of total phenols decreases as the S/L increases, which means that the polyphenols extracted from olive leaves were diluted since higher solvent volume was used. Moreover, the extraction yield (mg TP/g d.w. olive leaves) increased up to 1/8 S/L ratio and remains constant till 1/10 S/L. Thus, from an economical point of view a compromise has to be met between a relatively high extraction yield and a relatively high polyphenols concentration in the extract. The optimum condition that fulfils this requirement is at that S/L value in which the curves of extraction yield and concentration of total phenols are crossed each other. Thus, a solid-liquid ratio 1:7 is accordingly recommended. This ratio is similar to the value that Bilek [27] suggests for optimum extraction conditions for total phenolic compounds from dried olive leaves based on the response surface methodology analysis.

### 3.4. Effect of Ethanol Concentration

Several solvent systems have been used to recover phenolic compounds from plant matrices. Ethanol has been classified as generally recognized as safe (GRAS) as well as has been reported to be an effective solvent for the recovery of phenolic compounds and thus, is usually used to recover this group of phytochemicals, especially when it comes to the production of nutraceuticals [28]. Some authors reported that the effectiveness of the phenolic compound recovery through solvent extraction with ethanol can be increased by the addition of different proportions of water [29,30]. Figure 1b demonstrates the effect of ethanol-water composition on total phenols content with the highest value being obtained at 70% (v/v) aqueous ethanol concentration. Studies dealing with the extraction of polyphenols from olive leaves have recommended different proportions of ethanol-water varied at range of 40%–80% v/v [14,15,19]. This is due to the fact that there is not a single ethanol-water portion that can extract effectively all the phenolic compounds present in different plant matrices [31] and hence it needs to be carried out on a case-by-case basis.

### 3.5. Multistage Extraction of Olive Leaves

**Figure 2:**
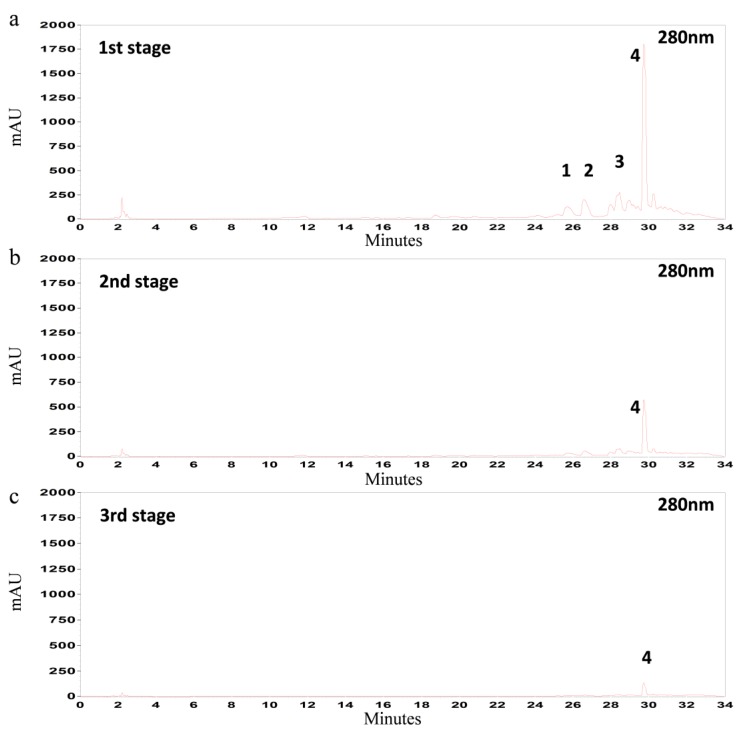
Chromatographic analysis (HPLC-DAD) of ethanolic olive leaf extract at 280 nm. (**a**) first stage; (**b**) second stage; and (**c**) third stage. **1**: luteolin-7-*O*-glucoside; **2**: verbascoside; **3**: apigenin-7-*O*-glucoside; and **4**: oleuropein.

Most of the studies dealing with the optimization of the extraction of polyphenols have either followed protocols with long extraction times (5–48 h), often at elevated temperatures (˃40 °C), or have used sophisticated techniques which may have given higher extraction yields and shorter extraction times but are still suffering from high energy consumption as they operate, e.g., under high pressures. Thus, a new procedure is required which lies between the technical simplicity of the traditional extraction methods and the high efficiency of the advanced techniques. Following this principle, thermal pre-treatment (blanching) of olive leaves and multistage extraction were combined from moderate to high temperatures. In many studies, the extraction efficiency has been mainly evaluated with determination of total phenol content using Folin-Ciocalteau assay. However, the use of this reagent for quantification of total phenols has some drawbacks. The current reagent interacts with the free hydroxyl groups of phenolic compounds via electron transfer mechanism. These hydroxyl groups can be formed either by cleaving high molecular weight polyphenols (e.g., oleuropein) to simple ones (e.g., hydroxytyrosol) or by cleaving the sugar moiety resulting in the formation of aglycones of phenolic compounds. Moreover, the phenolic compounds have different reactivity with Folin-Ciocalteau reagent [32] which can be reduced by many non-phenolic compounds; e.g., vitamin C, Cu(I), [33] resulting in overestimations or underestimations in the total phenol content. Thus, optimization of extraction of phenolic compounds from olive leaves, applying multistage extraction scheme, has to be carried out based on HPLC-DAD analysis (Figure 2) of the main phenolic compounds present in olive leaf extract at each stage of extraction.

**Figure 3:**
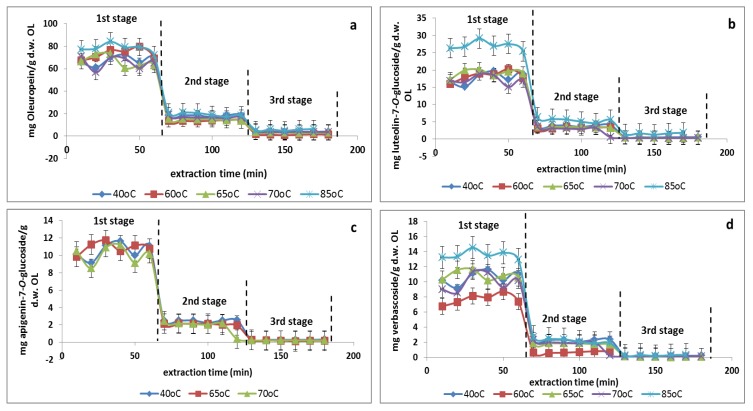
Mean values (*n* = 3) of extraction yields (mg/g d.w. OL) of (**a**) oleuropein; (**b**) luteolin-7-*O*-glucoside; (**c**) apigenin-7-*O*-glucoside; and (**d**) verbascoside which are obtained after multistage extraction (60 min/stage) (sampling was performed every 10 min) at 40, 60, 65, 70 and 85 °C. The extraction was performed at pH 2.5 with solid-to-liquid (S/L) ratio 1:7, 70% aqueous ethanol solution and 0.5 mm particle size of OL.

Figure 3 illustrates the yields (mg/g d.w. OL) of oleuropein (Figure 3a), luteolin-7-*O*-glucoside (Figure 3b), apigenin-7-*O*-glucoside (Figure 3c) and verbascoside (Figure 3d) at each temperature for every stage of the extraction of steamed olive leaves. The first observation is that the amount of polyphenols that has been extracted at the first 10 min does not differ significantly from the amount extracted at the last 60 min of each stage. This principle applies for every stage at each temperature and for all phenolic compounds. Hence, the duration time of each stage does not need to be more than 10 min. Consequently, the total duration time which is enough for sufficient recovery of polyphenols, should not be more than 30 min (10 min × 3 stages); provided that the olive leaves have been previously steam blanched. Regarding to the performance of the multistage extraction, at the 1st stage 75.5% ± 2.8% of the oleuropein, 80.1% ± 3.2% of the luteolin-7-*O*-glucoside, 84.4% ± 3.9% of the verbascoside and 80.4% ± 2.2% of the apigenin-7-*O*-glucoside had been extracted from olive leaves.

The highest total extraction yield (166.6 ± 0.9 mg/g d.w. OL) was obtained at 85 °C compared to 70 °C (140.3 ± 0.9 mg/g d.w. OL), 65 °C (133.0 ± 0.8 mg/g d.w. OL), 60 °C (133.5 ± 1.0 mg/g d.w. OL) and 40 °C (137.5 ± 0.7 mg/g d.w. OL), as presented in Table 1. Based on the results, there was only an 18% increase of the extraction yield when the temperature was raised by more than 2-fold (40–85 °C). Therefore, from energetic and hence from economical point of view, this relative improvement on the extraction yield does not justify the operation of extraction at high temperatures (>40 °C). Consequently, the combination of thermal pre-treatment and multistage extraction provides moderate temperatures and short extraction times for a satisfactory recovery of phenolic compounds from olive leaves. However, the behavior of each polyphenol was different regarding temperature fluctuations. It is observed that there was an impact on the extraction yield of luteolin-7-*O*-glucosed (Figure 3b) and verbascoside (Figure 3d) when multistage extraction was operated at different temperatures compared to oleuropein (Figure 3a) and apigenin-7-*O*-glucoside (Figure 3c). The total yield of luteolin-7-*O*-glucoseide and verbascoside was 33% and 18% higher (Table 1) respectively, when a 48% increase in operation temperature was applied. Nevertheless, these differences in extraction yields of each polyphenol contribute only 18% on the overall extraction yield increment.

In the case of multistage extraction at 40 °C of non-steamed olive leaves, the total yield of oleuropein was 6.5 mg/g d.w. OL compared to 4.6 mg/g d.w. OL when single extraction step was applied. Moreover, the yields of oleuropein obtained by multistage (3 stages) and single step extraction at 40 °C of steamed olive leaves, were 89.5 mg/g d.w. OL and 47.7 mg/g d.w. OL, respectively. Thus, the combination of steam blanching and multistage extraction can significantly increase the yield of about 48% at short extraction times, *i.e.*, 30 min instead of 48 h. The yield of oleuropein was further increased at 85 °C (103.5 mg/g d.w. OL) resulting in an additional 6% increase. As mentioned before, this small increase does not justify the need for operating at such of high temperatures which consequently leads to an energy-intensive extraction process.

**Figure 4:**
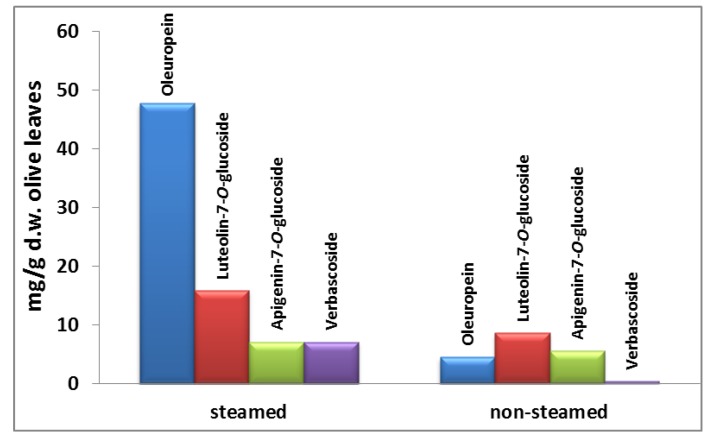
Total extraction yields of the main polyphenols which were determined in OL extract of steamed and non-steamed OL with HPLC-DAD after conventional extraction (70% ethanol, 48 h under stirring 400 rpm at 40 °C).

Examining the effect of steam blanching on the extractability of phenolic compounds during single step extraction, a highly significant increase of oleuropein (10-fold) and verbascoside (14-fold) yields was observed, compared to luteolin-7-*O*-glucoside (2-fold) and apigenin-7-*O*-glucoside (1.3-fold) (Figure 4). These dissimilarities between the polyphenols yields could be due to their different location within plant tissues [34] as well as their interaction with other plant constituents [31]. Thus, the effectiveness of the extraction seems to depend mainly on the diffusibility of the organic solvent within plant tissue when the composition of the solvent remains constant. This could be supported by the fact that the extraction yield of oleuropein from steamed olive leaves was further increased 2-fold when multistage extraction was applied, *i.e.*, improvement of the mass transfer coefficient, without changing solvent composition and temperature.

The total amount of phenolic compounds which has been obtained from single extraction of steam blanched olive leaves was 77.92 mg/d.w. OL whereas the total amount of phenolic compounds from non-steam blanched olive leaves was 19.23 mg/g d.w. OL (Figure 4) which means a 75% increase in extraction yield.

**Table 1:** Polyphenols composition in olive leaf extract at each extraction stage performed at different temperatures. The values in parenthesis represent the corresponding percentage of phenolic compound in the olive leaf extract at every stage. * Mean values (mg/g d.w. OL) of each phenolic compound at every stage (*n* = 6, ±SD).

Temperature	Extraction Stage	Total	Oleuropein *	Luteolin-7-*O*-glucoside *	Verbascoside *	Apigenin-7-*O*-glucoside *
85 °C	1st	129.8 ± 1.8	78.2 ± 2.9 (60.2)	27.0 ± 1.2 (20.7)	13.5 ± 0.5 (10.3)	11.1 ± 0.9 (8.5)
	2nd	29.3 ± 0.9	19.4 ± 1.2 (66.1)	5.5 ± 0.6 (18.8)	2.2 ± 0.3 (7.5)	2.2 ± 0.5 (7.5)
	3rd	7.5 ± 0.2	5.5 ± 0.3 (72.6)	1.2 ± 0.2 (16.7)	0.3 ± 0.1 (3.9)	0.5 ± 0.1 (6.6)
	*Total*	166.6 ± 0.9	103.1 ± 1.5	33.7 ± 0.6	16 ± 0.9	13.8 ± 0.5
70 °C	1st	103.5 ± 2.0	65.4 ± 4.3 (63.1)	17.1 ± 1.4 (16.5)	9.9 ± 1.2 (9.5)	11.1 ± 1.0 (10.7)
	2nd	29.3 ± 0.7	16.9 ± 0.4 (57.6)	3.1 ± 1.1 (10.8)	2.0 ± 0.7 (6.8)	2.2 ± 0.7 (7.5)
	3rd	7.5 ± 0.1	3.9 ± 0.1 (51.5)	0.9 ± 0.1 (4.7)	0.7 ± 0.1 (1.8)	0.5 ± 0.1 (6.6)
	*Total*	140.3 ± 0.9	86.2 ± 1.6	21.4 ± 0.8	12.6 ± 0.6	13.8 ± 0.6
65 °C	1st	107.4 ± 2.0	66.5 ± 5.5 (61.9)	19.1 ± 1.1 (17.7)	10.9 ± 0.6 (10.1)	10.9 ± 0.6 (10.1)
	2nd	21.9 ± 0.2	14.6 ± 0.5 (66.6)	3.4 ± 0.1 (15.5)	1.8 ± 0.1 (8.2)	2.1 ± 0.1 (9.5)
	3rd	3.7 ± 0.1	3.04 ± 0.2 (81.9)	0.4 ± 0.1 (11.0)	0.6 ± 0.1 (1.6)	0.4 ± 0.1 (5.3)
	*Total*	133.0 ± 0.8	84.1 ± 2.1	23.3 ± 0.4	13.3 ± 0.3	4.5 ± 0.3
60 °C	1st	109.9 ± 2.6	73.1 ± 4.8 (66.5)	18.2 ± 4.2 (16.5)	7.7 ± 0.7 (7.0)	10.9 ± 0.8 (9.9)
	2nd	20.9 ± 0.2	13.7 ± 0.3 (65.5)	3.3 ± 0.1 (15.7)	1.8 ± 0.1 (8.6)	2.1 ± 0.2 (10.0)
	3rd	2.7 ± 0.1	1.5 ± 0.4 (55.6)	0.5 ± 0.1 (19.7)	0.5 ± 0.1 (18.2)	0.2 ± 0.1 (7.2)
	*Total*	133.5 ± 1.0	29.4 ± 1.8	22 ± 1.5	10 ± 0.3	13.2 ± 0.4
40 °C	1st	105.9 ± 1.9	67.3 ± 4.1 (63.5)	17.6 ± 1.6 (16.6)	10.5 ± 0.8 (9.9)	10.5 ± 0.9 (9.9)
	2nd	27.0 ± 0.2	18.3 ± 0.7 (67.7)	3.9 ± 0.2 (14.4)	2.3 ± 0.1 (8.5)	2.5 ± 0.1 (9.2)
	3rd	4.6 ± 0.1	3.9 ± 0.1 (84.7)	0.2 ± 0.1 (4.3)	0.2 ± 0.1 (4.3)	0.3 ± 0.1 (6.5)
	*Total*	137.5 ± 0.7	89.5 ± 1.6	21.7 ± 0.6	13.0 ± 0.3	13.3 ± 0.4

### 3.6. Antioxidant Activity of Ethanolic Extracts of Olive Leaves

**Figure 5:**
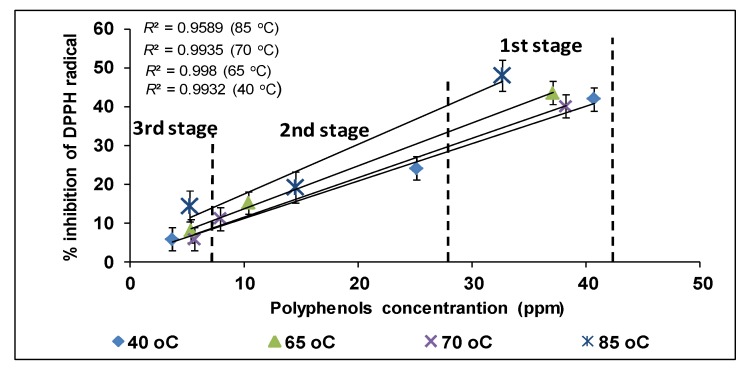
Antioxidant activity of ethanolic extracts of olive leaves. * Sum of the oleuropein, verbascoside, luteolin-7-*O*-glucoside and apigenin-7-*O*-glucoside concentrations at the end of each stage of the multistage extraction which had been performed at 40, 65, 70 and 85 °C and dissolved prior to the addition in DPPH (2,2,-diphenyl-2-picryl-hydrazyl) solution. Mean values (*n* = 3, ±SD).

The antioxidant activity of ethanolic extracts of olive leaves was followed a linear relationship with polyphenols concentration (Figure 5). Nevertheless, there were variations between the samples of different experimental conditions. Particularly, the sample of the 1st stage at 85 °C with concentration 32 ppm gave higher inhibition value (48%) compared to 42% inhibition value of the sample (41 ppm) which was collected from the 1st stage at 40 °C (Figure 5). The low correlation of polyphenols and antioxidant activity has been related to the different phenolic composition of ethanolic extracts (Table 1; values in parenthesis) [22] and to the antagonistic and synergistic phenomena in mixtures of pure polyphenols and OL extract [35]. The antioxidant activity of ethanolic extracts obtained after multistage extraction of steamed olive leaves was 10 times higher than the ethanolic extracts obtained with the conventional extraction method.

## 4. Conclusions

The most important highlights of the present study are as follows:

The current study showed that the combination of steam blanching process and multistage extraction (after optimization) is advantageous since it provides short extraction times (≤30 min) at moderate operation temperatures (40 °C).

The improvement of extraction yield that can be achieved is more than 50% compared to the conventional extraction method.

The antioxidant activity of ethanolic extract of steamed olive leaves which had been extracted by the multistage process was 10 times higher than that obtained by the conventional extraction method.

The current extraction scheme does not require sophisticated or complicated techniques.
